# Clinical research for whether the Traditional Chinese medicine could promote the resorption of lumbar disc herniation: a randomized controlled trial

**DOI:** 10.1097/MD.0000000000021069

**Published:** 2020-07-02

**Authors:** Jintao Liu, Yu Zhu, Zhiqiang Wang, Pengfei Yu, Chunchun Xue, Hong Jiang, Xiaofeng Li, Dezhi Tang

**Affiliations:** a Suzhou Hospital of Traditional Chinese Medicine, Suzhou, Jiangsu; b Longhua Hospital, Shanghai University of Traditional Chinese Medicine, Shanghai; c Shanghai Traditional Chinese Medicine Hospital, PR China.

**Keywords:** Japanese Orthopedic Association score, lumbar disc herniation, magnetic resonance imaging, randomized controlled trial, spontaneous resorption, traditional Chinese medicine, visual analog scale

## Abstract

Lumbar disc herniation (LDH) is a common, disabling musculoskeletal disorder. Magnetic resonance imaging has clarified the natural history of lumbar disc lesions and has documented that disc lesions can become smaller and can even be completely resorbed. Previous studies have confirmed that some traditional Chinese medicine (TCM) therapies can promote resorption of the protrusion. However, high-quality research evidence is needed to support the effectiveness of the protocol.

**Objective:**

This clinical trial aims to establish whether TCM can promote the resorption of LDH and to assess the efficacy of such therapy for LDH, thereby evaluating its clinical effect.

**Methods:**

The present study design is for a single-center, 2-arm, open-label randomized controlled trial. A total of 150 eligible LDH patients will be randomly assigned to either a TCM treatment group or a control group in a 1:1 ratio. Patients in the TCM group will be administered a TCM decoction for 4 weeks. Patients in the conventional drug control group will be instructed to take a specific daily dose of celecoxib. The primary outcome measure is the change from baseline in the volume of the protrusion, as assessed using MR images. Secondary outcome measures include visual analog scale pain scores and Japanese Orthopaedic Association scores assessed at 3 and 6 months.

**Discussion:**

The design and methodological rigor of this trial will allow evaluation of the basic clinical efficacy and safety data for TCM in the treatment of patients with LDH. The trial will also assess whether TCM can promote the resorption of LDH. This research will therefore help provide a solid foundation for the clinical treatment of LDH and for future research in TCM therapy.

**Trial registration:**

ChiCTR1900022377.

## 1 Introduction

Lumbar disc herniation (LDH) is a common disease leading to lower back pain and neurological symptoms, including radiating pain in the lower extremities.^[1]^ After other diseases have been ruled out, LDH is found in approximately 85% of patients suffering from lower back pain and radiculopathy.^[2]^ The annual incidence of LDH is 5 per 1000 adults.^[3]^ About 60-90% of LDHs can be treated conservatively alone.^[4]^ Patients with obviously extruded discs or marked neurological deficits can be even successfully treated with the active conservative treatment.^[5]^

In 1984, Guinto et al. first reported a case of lumbar disc degeneration after conservative treatment.^[6]^ Thereafter, many other researchers have reported similar findings regarding spontaneous regression of LDH.^[7–11]^ Many studies have assessed the natural history of lumbar disc lesions. Such studies, based on magnetic resonance imaging (MRI) and computed tomography (CT)data, have documented that disc lesions can become smaller and can even completely resolve.^[9,12,13]^ The phenomenon of LDH reabsorption is now recognized, and its overall incidence has reached 66.66% of LDH cases, according to a meta-analysis.^[14]^

In China, traditional Chinese medicine (TCM) is 1 of the main conservative treatments for LDH. Some studies have confirmed that some TCM therapies has a certain therapeutic effect on lumbar back pain caused by lumbar disc herniation and stimulates the resorption of herniated disc.^[9,15–17]^ However, the limited quality and small number of subjects of the few trials that have been conducted have made it difficult to reach firm conclusions about these treatments. High quality research evidence is needed to support the effectiveness of TCM regimens. This study protocol was created to investigate whether TCM can promote the resorption of LDH, to judge the efficacy of TCM therapy for LDH, and to evaluate its clinical effects.

## 2 Methods

### 2.1 Study design

This clinical trial will be a randomized, controlled trial. Subjects will be enrolled at Suzhou Hospital of TCM. The Ethics Boards of Suzhou Hospital of TCM have approved this study. All study participants will give written informed consent before participation.

### 2.2 Ethics approval and consent to participate

The study is in compliance with the Declaration of Helsinki. Only clinicians holding the necessary qualifications are acting as principal investigators. The final amendments (version: April 06, 2019) and the consent form have been reviewed and approved by the Ethics Committee of Suzhou hospital of TCM (reference number: SZTCM2019-4-01). If there is any amendment to the protocol, approval must be again sought from the Ethics Committee. Written informed consent will be obtained from each participant before enrolment.

### 2.3 Inclusion criteria

(1) In this trial, we refer to the literature^[9,15–17]^ and set the inclusion criteria as follows: (1) age 20 to 60 years;
(2) pain intensity ≥40 mm on the 100-mm pain visual analog scale (VAS) for lower back pain;
(3) having low back pain and/or radiating pain from the lower extremities caused by LDH (MRI scan confirmed lumbar disk herniation in our hospital in the previous 2 weeks);
(4) willing to participate in this study and giving informed consent;
(5) written consent provided to participate in 2 follow-up MRI scans (at 3 months and 6 months).

### 2.4 Exclusion criteria

In this trial, we refer to the literature^[9,15–17]^ and set the exclusion criteria as follows:

(1) red flag signs that may indicate cauda equina syndrome, such as bladder and bowel dysfunction or saddle anesthesia;
(2) a history of spinal surgery;
(3) having serious chronic diseases that could interfere with the outcomes;
(4) pregnant or planning to become pregnant during the study;
(5) having other diseases that the researchers believe render the subject unsuitable for the study;
(6) patients whose MRI scans were low resolution or unsuccessful.

### 2.5 Recruitment

Participants will be recruited through advertisements in local newspapers, bulletin boards, and on the websites of local medical centers. All patients will be screened initially to establish a baseline assessment of selection criteria. If the inclusion criteria are met and the informed consent form is signed, the patient will be submitted for randomization.

### 2.6 Randomization

In this trial, participants will be randomly assigned to either the TCM group or the control group in a 1:1 ratio using a random number generator (SPSS 16.0, SPSS Inc, Chicago, IL).

### 2.7 Blinding

Because the study will be an open-label clinical trial, both patients and clinicians will know which treatment is being administered, and patients are required to cooperate with their physicians prior to treatment. The assessment of clinical efficacy will be carried out by telephone by a clinical assessor who will be masked to the treatment assignment. During the data collection and analysis stages, the clinical researchers, assessors, and statisticians will not share study information.

### 2.8 Intervention

**Figure 1:**
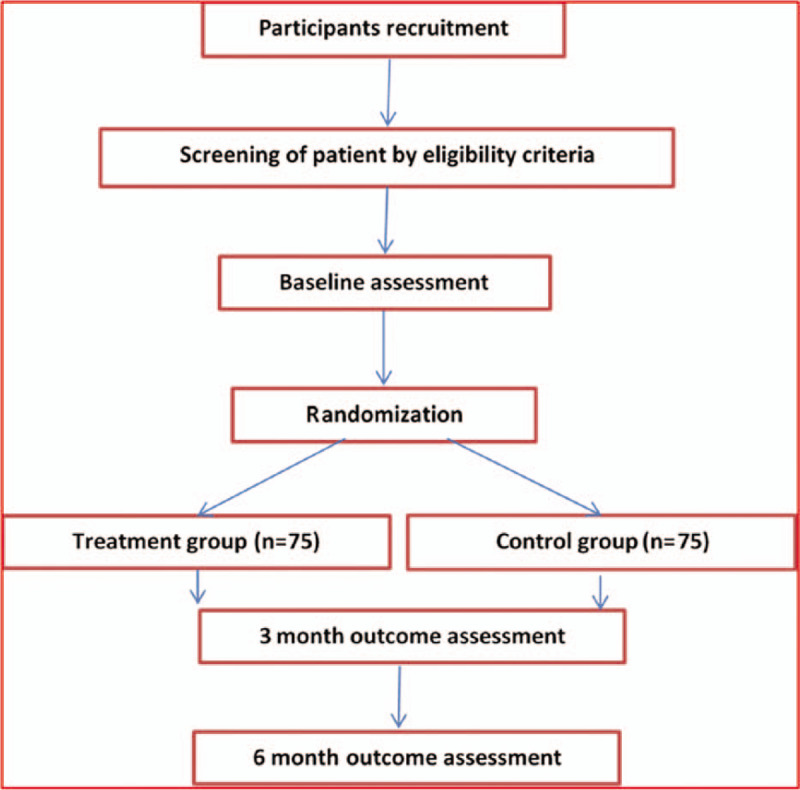
Flowchart for participant identification, inclusion, study design, interventions, assessments, and follow-up.

This study will be conducted in accordance with the requirements outlined in the Declaration of Helsinki, and with the approval of the appropriate Institutional Review Boards. Each participant will sign the written informed consent form before undergoing any examination or study procedure, in compliance with Good Clinical Practice. Eligible patients will be randomized into 1 of the 2 groups: the TCM treatment group and the control group. Follow-up will be carried out by telephone inquiry and outpatient reexamination, which will be performed 3 and 6 months after the first visit. VAS and Japanese Orthopedic Association (JOA) scores will be recorded at each follow-up visit. MRI investigations will be performed on the first visit and at the 3-month and final follow-up to calculate the volume of the protrusion (Fig. 1).

### 2.9 TCM treatment group

(1) Absolute bed rest is required for 4 weeks. Once permitted to ambulate, the patient is to wear a girdle for 4 to 8 weeks.
(2) The patient is to take the following TCM preparation: (raw *Astragalus* 20 g, roasted *Astragalus* 20 g, Radix *Stephaniae tetrandrae* 10 g, *Angelica sinensis* 10 g, *Ligusticum wallichii* 10 g, Rhizoma *Atractylodis macrocephalae* 10 g, *Lumbricus* 10 g, leech 6 g, Radix Clematidis 10 g, pawpaw 10 g, and *Brassica alba* Boiss. 6 g decocted in 500 ml water and taken orally at a dosage of 300 mL/d for 4 weeks.
(3) The patient is to perform the following exercises for 12 to 24 weeks:
  I. 20 to 30 repetitions of 5-point support, in which the patient assumes a supine position and raises the abdomen and pelvis as high as possible for 3 s while supporting the body with the head, elbows, and feet. These actions are to be carried out 3 times a day.
  II. 10 to 20 repetitions of swallow style, in which the patient assumes a prone position with the hands behind the back and raises the head and chest for 3 to 5 s while pushing the thighs back. These actions are carried out 3 times a day.

### 2.10 Control group

Patients in the control group receive 4 weeks of normal conservative treatment. Intervention measures fall into 4 sections:

(1) Health education. Patients are invited to receive LDH health education twice a week in an outpatient setting; the health education is designed exclusively to inform patients about the natural course of their illness and the expectation of successful recovery, irrespective of the initial intensity of their pain. Patients will be educated to avoid some bad habits that aggravate the disease, such as remaining in the sitting position for a long time and carrying heavy loads. Patients are encouraged to participate in social activities.
(2) Absolute bed rest is required for 4 weeks. Once ambulation is permitted, the patient is to wear a girdle for 4 to 8 weeks.
(3) For the first 4 weeks, if the pain is not relieved, the patient may take 0.1 g b.i.d. of celecoxib.
(4) The patient is to perform the following exercises for 12 to 24 weeks:
  1. 20 to 30 repetitions of 5-point support, in which the patient assumes a supine position and raises the abdomen and pelvis as high as possible for 3 s while supporting the body with the head, elbows, and feet. These actions are carried out 3 times a day.
  2. 10 to 20 repetitions of swallow style, in which the patient assumes a prone position with the hands behind the back and raises the head and chest for 3 to 5 seconds while pushing the thighs back. These actions are repeated 3 times a day.

### 2.11 Indications for surgery

Surgery will be indicated if:

(1) after the termination of all interventions, conservative treatment with the TCM regimen for 3 to 6 months has been ineffective (Japanese Orthopaedic Association scores <16); or
(2) the patient experiences exacerbation or progression of radicular symptoms or cauda equina neurological signs at any stage during the treatment period.

### 2.12 Outcome measures

#### 2.12.1 Primary outcome measure

##### 2.12.1.1 Changes in protrusion size

**Figure 2:**
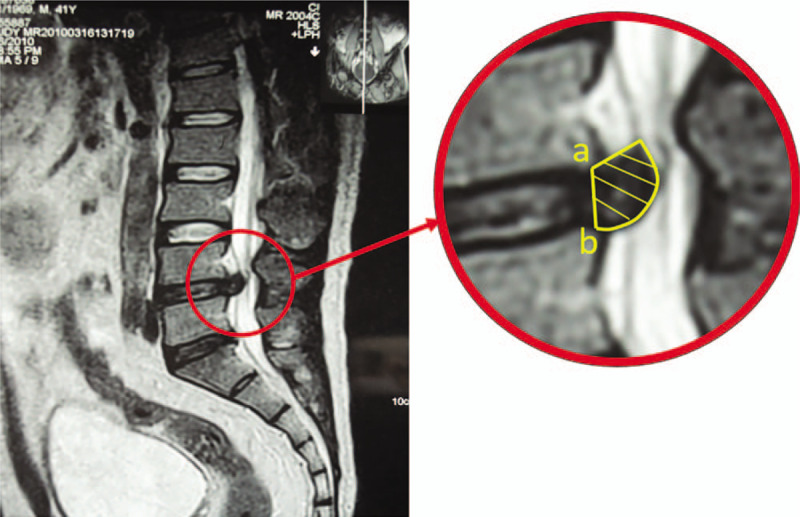
Representative MR image to calculate the resorption rate. MR = magnetic resonance.

MR images were assessed to measure changes in protrusion size. A 1.5-T MRI system (Siemens, Erlangen, Germany) was used with a spin-echo sequence. Eleven sections were scanned on T1- and T2-weighted sagittal views with an interlamellar spacing of 1.25 mm and a section thickness of 5 mm. The image data were scanned and processed by Picture Archiving and Communication Systems. The volume and resorption rate of the protrusion were calculated according to the method described by Autio.^[18]^ MRI scans were performed on the first visit, the second visit, and the final follow-up to calculate the volume of the protrusion (ie, baseline and 3- and 6-month follow-up). T2-weighted sagittal view MR image showing how the resorption rate is calculated.^[16]^ The internal boundary of the protrusion is the line connecting the posterior inferior margin of the upper centrum and the posterior superior margin of the lower centrum. The external boundary is the protrusion edge. A proficient MRI operator can determine the area of the protrusion, as shown in the right panel. The volume of the protrusion (mm^3^) = (inter-section spacing + section thickness) (mm) ×Σ area of the protrusion in each section (mm^2^). The resorption rate = (volume of protrusion before treatment—volume of protrusion after treatment/volume of protrusion before treatment) ×100(%). Representative images are shown in Figure 2.

### 2.13 Secondary outcome measures

#### 2.13.1 VAS pain score

The primary efficacy endpoint of the study will be the VAS, which is a pain score ranging from 0 mm (no pain) to 100 mm (worst pain ever experienced). Operationally, the VAS score is usually a horizontal line, 100 mm in length, anchored by word descriptors at each end. Patients mark the point on the line representing the current pain level. The VAS score is then determined by measuring in millimeters from the left-hand end of the line to the point that the patient marked.^[19]^ The VAS score will be measured during each assessment visit (at baseline and 3-and 6-month follow-up)

#### 2.13.2 JOA score

The JOA score indicates the severity of clinical symptoms. The improvement in JOA score is calculated according to the following equation:^[16]^ Improvement according to JOA score (%) = (score after completion of treatment − score before treatment)/ (29 − score before treatment) × 100 (the maximum possible score is 29). An improved JOA score ≥75% was classified as excellent, 50% to 75% as good, 25% to 50% as fair, and <25% as poor. The JOA score will be measured during each assessment visit (ie, at baseline and 3- and 6-month follow-up).

#### 2.13.3 Sample size calculation

The required sample size was calculated using G∗Power 3 software, developed by the Institute for Experimental Psychology (Heinrich-Heine University, Germany). For this trial, it was determined prospectively that α =0.05 and 1-β =0.90. Consistent with a previous trial on massage for LDH, a total of 150 participants will be included in this trial (75 in each group) to compensate for an anticipated dropout rate of 15%.

### 2.14 Data analysis

Statistical analysis will be performed using SPSS 16.0. Measured data such as JOA scores and the volume of the protrusion will be compared using Student *t-*test for matched data; whereas enumerated data, such as the improvement in the JOA score, will be compared using the chi-squared test or Fisher exact probability test. *P* <.05 will be considered significant for all statistical tests.

## 3 Discussion

LDH commonly occurs in patients 20 to 40 years of age and results in acute symptoms of shooting and intractable pain in the low back and/or lower extremities.^[20]^ However, the prognosis of these patients is usually very good. Moreover, 70% of LDH patients have been reported to be free from sciatica at approximately 6 months after the first onset.^[20]^ Ultimately, conservative treatment may be the best option when radiculopathy is acceptable and cauda equina syndrome is absent.^[14,21]^

MRI studies have clarified the spontaneous resorption process of herniated discs, which is a major cause of the reduction of symptoms in LDH patients.^[20,22]^ Recent advances in MRI techniques have facilitated the examination of nerve tract fibers and the identification of symptomatic nerve tissue. The LDH resorption process has been demonstrated using sequential MRI, and this resorption process may be the reason for the relatively good prognosis in cases of LDH.^[23]^

Mechanisms relating to spontaneous disc regression have been described in the literature. The first mechanism used to explain spontaneous disc regression is that the signal intensity of the nucleus pulposus at the initiation of the hernia is higher than that in the original nucleus, and then decreases with time. The hydration of the nucleus pulposus at the initiation of the herniation is then rapidly followed by dehydration.^[24]^ The second mechanism is the resorption theory. The nucleus pulposus with hernia is considered to be a foreign body by the immune system, and the inflammatory process starts with neovascularization. The nucleus pulposus with hernia is then eliminated by enzymatic catabolism and phagocytosis.^[25,26]^ The third mechanism proposes that enzymatic degradation and phagocytosis of cartilaginous tissue occurs as a result of the disc regression inflammatory reaction and neovascularization of disc herniation.^[27,28]^ The vascular supply may play a major role in resorption of the disc material. The vascular mechanism of resorption involves a local reaction around the disc fragments, the proliferation of blood vessels, and the migration of the phagocytes to the disc material; matrix proteinases and increased cytokine levels play a role in the spontaneous regression process.^[29,30]^ If the above mechanisms can be activated through drugs, in particular TCM, this will undoubtedly both alleviate the suffering of patients and increase social benefits.

Some studies have reported the effectiveness of herbal medicine, which is the most popular form of complementary and alternative medical therapy for the treatment of LDH, either used alone or concomitantly with usual care.^[9,15,16]^ The limited quality and small sample sizes of the few trials that have been conducted to date have made it difficult to reach firm conclusions about TCM treatments. Well-designed randomized controlled trials are needed to examine the efficacy of TCM treatments for LDH. The purpose of the study planned here is to evaluate the basic clinical efficacy and safety data of TCM in the treatment of patients with LDH. The study should also help to establish whether TCM promotes the resorption of LDH.

## Author contributions

**Conceptualization:** Jintao Lliu, Yu Zhu and Zhiqiang Wang

**Data curation**: Pengfei Yu, Chunchun Xue

**Formal analysis**: Chunchun Xue, Xiaofeng Li

**Investigation**: Hong Jiang, Zhiqiang Wang

**Methodology**: Pengfei Yu, Dezhi Tang

**Project administration**: Jintao Lliu, Hong Jiang

**Resources**: Jintao Lliu, Hong Jiang, Zhiqiang Wang

**Software**: Chunchun Xue, Zhiqiang Wang

**Supervision**: Xiaofeng Li, Dezhi Tang

**Validation**: Jintao Lliu, Yu Zhu

**Visualization**: Pengfei Yu, Zhiqiang Wang

**Writing – original draft**: Chunchun Xue, Xiaofeng Li

**Writing – review & editing**: Jintao Lliu, Yu Zhu, Zhiqiang Wang
